# Correlation between micro-hardness and mineral content in healthy human enamel

**DOI:** 10.4317/jced.53345

**Published:** 2017-04-01

**Authors:** Anna Akkus, Daniel Karasik, Renato Roperto

**Affiliations:** 1 Department of Comprehensive Care, Case Western Reserve University, Cleveland, Ohio 44106, USA; 2 University of Chicago, Hyde Park, Illinois 60637, USA

## Abstract

**Background:**

Enamel is the hardest and the stiffest tissue in the human body. The enamel undergoes multidirectional stresses, withstands multimillion chewing cycles, all while protecting the internal dentin and pulp from damage due to mechanical overload and exposure to the harsh chemical environment of the mouth. Raman spectroscopy allows to study enamel mineral content in a non-destructive and site-specific way. While Raman spectroscopy has been applied in other studies to assess tooth mineralization, there are no studies that examine the relationship between micro-hardness and mineral content of the untreated enamel. An understanding of this relationship is extremely important in a clinical context. The effect of various agents on enamel hardness was investigated, though the relationship between healthy enamel mineral content and micro-hardness remains obscure.

**Material and Methods:**

Twenty human incisor teeth were obtained in compliance with the NIH guidelines and imaged site-specifically with a Raman microscope and evaluated with a Brinell hardness measurement device. The front portion of each tooth was divided into apical, medium and cervical regions and subsequently imaged with a Raman microscope in these three locations.

**Results and Conclusions:**

The results demonstrated that enamel mineral content varies significantly between individuals and is correlated with the hardness of the enamel. Non-invasive, sample preparation free Raman spectroscopy was successfully employed to measure the mineral content of healthy enamel and it correlated the mineralization score to the hardness measurements of the selected cervical location. The overall level of enamel mineral content may serve as a robust predictor of patients’ susceptibility to developing caries, and overall enamels wear resistance, thus allowing for the prevention of caries via clinically available methods of remineralization, fluoride treatment and frequent cleaning.

** Key words:**Enamel, raman spectroscopy, micro-hardness, extracted teeth.

## Introduction

Enamel is the hardest and the stiffest tissue in the human body. Dental enamel is 95% mineral, 1% organic matter and 4-5% water by weight percentage (1). The enamel experiences multidirectional stresses and withstands multimillion chewing cycles, while protecting the internal dentin and pulp from damage due to mechanical overload and exposure to the harsh chemical environment of the mouth. Enamel mineralization is an important property that is positively correlated with the mechanical behavior of other tissues, such as bone (2) and teeth (3-5). Many dental ailments are caused by a reduction in enamel mineral content. For example, molar-incisor hypo mineralization increases tooth sensitivity to food, drinks, and thermal changes, and results in restoration failure (6,7). Moreover, previous studies suggest a possible link between enamel mineral concentration and susceptibility to caries (8,9).

Several authors have investigated enamel mineral content (10-14) using various characterization methods in the context of decay (1,6), demineralization/remineralization processes (15,16), age (17,18) and disease (19,20). The methods cited above, however, are limited to *ex vivo* laboratory conditions and are destructive to the specimens.

Raman spectroscopy offers the opportunity to study enamel mineral content *in vivo* (21), non-destructively and site-specifically. While Raman spectroscopy has been applied in the literature to assess tooth mineralization, there are no studies that examine the relationship between micro-hardness and mineral content of the untreated enamel. An understanding of this relationship is extremely important in a clinical context, specifically for tooth handling *in vivo* and structure property research inquiry of extracted teeth. It was found that enamel exposure to soft drinks adversely affects the hardness of the enamel and that this effect could be successfully counteracted via low concentration of iron supplement (22). Moreover (23) enamel from other biological species, such as sharks and crocodiles, were investigated and micro-hardness characteristics were found to be similar to that of human enamel. Although the effect of various agents on enamel hardness was effectively investigated, the relationship between enamel mineral content and micro-hardness remains obscure (24-26).

## Material and Methods

-Sample Preparation

**Figure 1:**
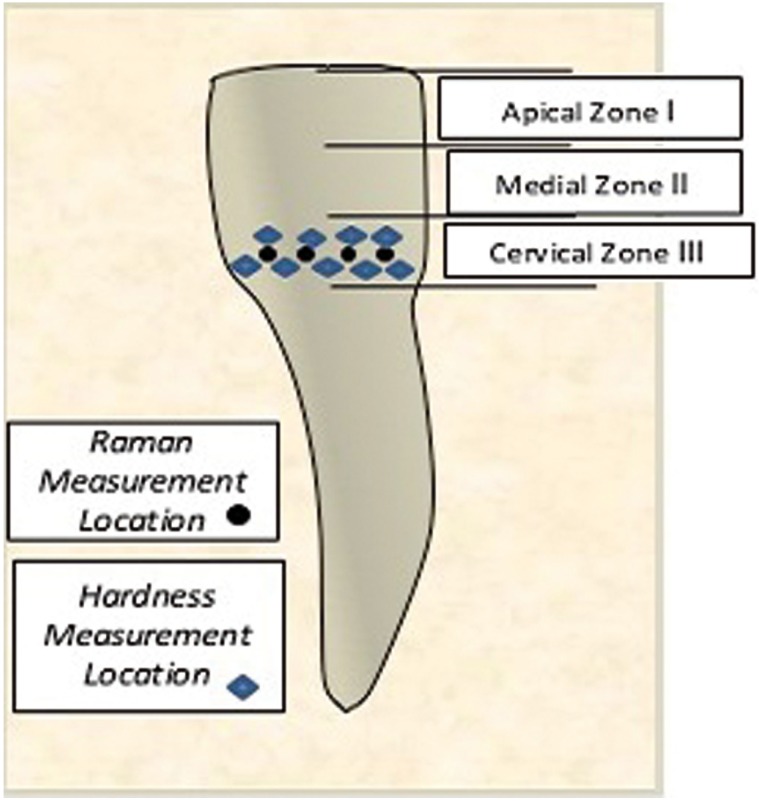
Schematic of the regions included in the Raman analysis and hardness measurements.

Human teeth were obtained in compliance with the National Institute of Health guidelines. The Institutional Review Board exemption was filed and approved (Protocol#: EM-13-17). Eleven adult human incisors were extracted as part of a normal treatment plan. The teeth were collected on the date of the extraction and kept moist at all times, without any additional disinfecting treatments. A dentist assessed the enamel of the specimens selected for Raman analysis in order to ensure that healthy, intact enamel was evaluated. The samples were wrapped in wet tissue paper individually and stored in a -20 ºC freezer. Each tooth was measured with a ruler and the length of the crown was divided into 3 zones along the y axis: apical, medial and cervical, as shown in figure 1. The cervical region was then evaluated. Prior to hardness measurements, the specimens were puttied in Whip Mix Mounding Plaster to ensure stability and horizontal, flat placement on the site of interest. Prior to the Raman analysis the specimens were thawed at room temperature for 30 minutes while being wrapped in moist tissue paper. The Raman measurements were taken in direct proximity of the indentation site from the nine hardness measurements (Fig. 1). A wet tissue paper was wrapped around the specimen to prevent dehydration during Raman scan, while exposing the region of interest for Raman analysis. Three measurements were taken within each zone in order to obtain the average mineralization within each zone.

-Raman Spectroscopy and Hardness Measurements

In Raman analysis, a laser light is focused on a sample using a lens or objective. The reflected photons carry information on the type and the amount of chemical bonds. In the case of mineralized tissues, the phosphate group in the mineral of the enamel strongly scatters Raman signals. Therefore, the amplitude of the phosphate peak in the Raman spectrum is proportional to the amount of mineral content. It is for this reason that Raman spectroscopy has long been a powerful tool for assessing mineral content, not only in the dentistry, but also other mineralized tissue, such as bone. In the current study, an 10x objective was used to focus the laser light. The resulting excitation spot was about 10 µm in diameter and the penetration of the laser was within 100 µm.

A Raman microscope (Labram Xplora, Horiba Jobin Yvon, Edison, NJ) with a laser source at 785 nm was used. Measurements were performed using a 1200 lines/mm grating, which provided a wavenumber resolution of 1.25 pixels/cm-1. Six acquisitions per point were taken. The Raman wavenumber shift measured by the system was calibrated using the known 520.7 cm-1 peak of a Si wafer. The mineralization was assessed based on the intensity of the 960 cm-1 peak of the phosphate (27) symmetric stretch band (1).

The specimens were positioned in Brinell hardness measurement device. Nine locations were measured under: 500g load was applied for 15 seconds (Fig. 1).

## Results

**Figure 2:**
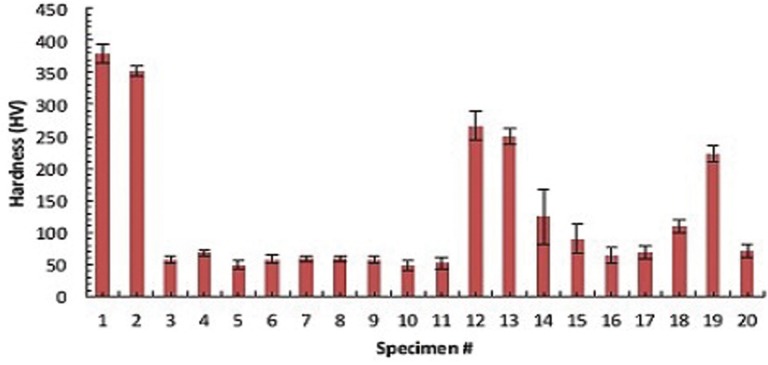
Hardness measurements for twenty human incisors in the cervical region of the crown.

**Figure 3:**
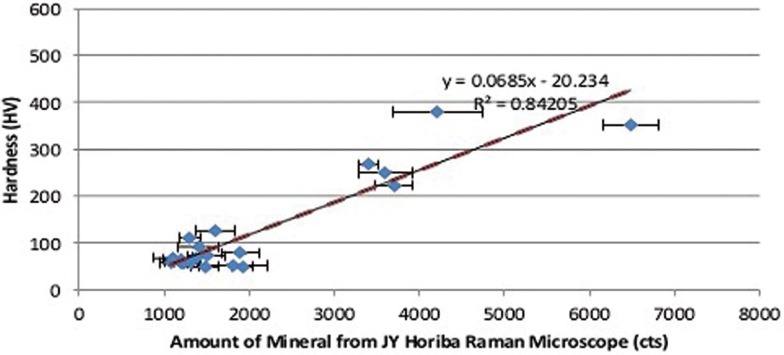
Correlation between hardness and mineral content in human enamel.

The height of the 960 cm-1 phosphate peak was measured to compare the mineral content levels in 20 incisors in the cervical area. The highest Raman-based mineralization intensity was about five times greater than the lowest measured mineral content (Fig. 2). The average value of enamel mineral content in the cervical region of the tooth was obtained by pooling together the measurements of the corresponding zone of 20 individuals with an average number of four measurements per tooth specimen. When the measurements were pooled within the cervical region, incisor hardness varied dramatically depending on the region (Fig. 3). Moreover a correlation between the amount of mineral and hardness was observed. The high hardness values corresponded to the high mineral content measurements obtained with the Raman microscope.

## Discussion

The results demonstrated that mineralization varies substantially between individuals and is correlated with the hardness of the enamel. In a previous study, it was also observed that the cervical enamel has the least mineralization with a populational average (28). The lower mineralization scores for the cervical region may be associated with a hygiene index. Dental plaque can normally be found on the cervical area of the tooth. Due to the highly acidic environment of the mouth, dental plaque can easily deactivate the enamel buffering capacity. Thus, it is important to understand the relationship between basic material properties and differences in degrees of mineralization in the cervical region of the tooth.

A range of values of mineral concentration has been reported (29-31) in previous studies (EDS, XRD analysis, X-ray microtomography). The methods employed, however, require destruction of the specimens, such as sectioning of the tooth into slices. Therefore, they are not clinically applicable. While computed tomography is viable, the spatial resolution of clinically available cone-beam systems is insufficient to capture the enamel layer. Subjection of the patient to ionizing radiation is another limitation. The Raman spectroscopy based assessment of mineralization holds a substantial promise for clinical application since Raman spectroscopy is noninvasive and does not require sample preparation. It is particularly informative in evaluating the mineral content of enamel due to the observed correlation between enamel hardness and overall measured mineral content in Raman microscope.

It has been suggested that the lower mineral concentration may be translated into increased porosity and is possibly linked to higher caries susceptibility. Moreover the results of this study demonstrate the lower hardness values for the enamel with lower mineral content score (32). Early identification of individuals with lower overall enamel mineralization may be a valuable way to begin to intervene before the development of caries.

## Conclusions

Non-invasive, sample-preparation free Raman spectroscopy was successfully employed to measure mineral content of healthy enamel and the measurements were used to find a correlation of the mineralization score to the hardness measurements of the selected cervical location. It was demonstrated that cervical region of the healthy enamel in the pool of the incisor specimens exhibit the lowest mineralization content in the cervical region of the crown. The overall level of enamel mineral content may serve as a robust predictor of patients’ susceptibility to developing caries, and overall enamels wear resistance, thus granting the opportunity to prevent the caries via clinically available methods of remineralization, fluoride treatment and frequent cleaning.
